# Factors Influencing the Intention of Chinese Adults to Recommend COVID-19 Vaccination for Specific or Non-Specific Groups

**DOI:** 10.3390/healthcare12141377

**Published:** 2024-07-10

**Authors:** Yuxin Pang, Bowen Li, Tongyao Li, Tianan Yang, Jianwei Deng, Wenhao Deng

**Affiliations:** 1 School of Management, Beijing Institute of Technology, Beijing 100081, China; 1120200918@bit.edu.cn (Y.P.); 3120221691@bit.edu.cn (B.L.); tianan.yang@bit.edu.cn (T.Y.); 111605@bit.edu.cn (J.D.); 2 Sustainable Development Research Institute for Economy and Society of Beijing, Beijing 100081, China; 3 Yangtze River Delta Research Institute, Beijing Institute of Technology, Jiaxing 314003, China; 4 Macquarie Business School, Macquarie University, Sydney, NSW 2109, Australia; tongyao.li@students.mq.edu.au

**Keywords:** COVID-19, vaccination, health literacy, vaccination hesitancy, China

## Abstract

The widespread availability of vaccines has profound implications for sustainable public health. Positive recommendation on vaccination is one of the most effective ways to increase the willingness to vaccinate against COVID-19. This study aims to investigate the factors influencing the intentions to recommend COVID-19 vaccination for specific groups (IRCVSG) and the intentions to recommend COVID-19 vaccination for non-specific groups (IRCVNSG) in China and explore the mediating role of vaccine hesitancy and perception of vaccination information. This study conducted a cross-sectional anonymous online survey of adults (N = 903) in 28 provincial-level administrative regions in China in May 2022. The prevalence of IRCVSG and IRCVNSG was 60.5% and 93.0%, respectively. Health information literacy has a significant direct and indirect impact on IRCVSG through safety hesitancy and the perceived adequacy and usefulness of vaccination information. The relationship between health information literacy and IRCVNSG is entirely mediated via hesitation about the effectiveness and perceived usefulness of vaccination information. Special attention should be paid to the safety hesitation of COVID-19 vaccination for specific groups. This study tests these effects from both theoretical and practical perspectives, helping to address barriers to promoting the vaccination of specific groups for COVID-19 in clinical practice, improving health and sustainability.

## 1. Introduction

As one of the most promising public health interventions for the prevention and control of the COVID-19 pandemic, continued progress on vaccination is critical for health and safety of people [1,2,3]. Specific groups (e.g., infants and children aged 6–23 months, pregnant women, older people over 60 years, and people with chronic and immune-compromising diseases) have generally low vaccination willingness and coverage, putting them at significant risk of infection and transmission [4,5,6,7]. Unlike non-specific groups, specific groups without intentions of receiving COVID-19 vaccination may be at high risk of widespread COVID-19 infection and also affect their cell-mediated immunity (CMI), resulting in extremely high rates of hospitalization, severe illness, and death [8,9,10,11].

Scholars have argued that subsidized vaccine costs and compulsory vaccination alone will not eliminate disease or increase vaccination and that social and public awareness and promotion of the COVID-19 vaccination play an essential role in improving vaccination coverage and protecting public health [12,13,14,15,16,17]. However, ignoring the specificity of the recommended population will make vaccination recommendations less effective and detrimental to promoting COVID-19 vaccination in specific groups [18,19]. Unfortunately, no current studies have focused on whether the intention to recommend vaccination to specific groups may differ from that of non-specific groups and whether the factors influencing the intention to recommend the COVID-19 vaccination to different populations differ. Therefore, exploring the current state and the factors that influence the intention to recommend COVID-19 vaccination to specific and non-specific groups will be vital in breaking the current bottleneck in vaccine coverage and successfully establishing a population-based immune barrier.

Based on the knowledge–attitude–practice (KAP) theory, this study aims to explore the behavioral intention and influencing factors of people’s recommendations of COVID-19 vaccines to specific and non-specific groups and build a mediating effect model through the two angles of knowledge and attitude to explore the influencing mechanism between health information literacy, vaccine hesitation, perceived vaccine information, and intentions to recommend COVID-19 vaccines.

## 2. Materials and Methods

### 2.1. Hypotheses Development

Based on the KAP theory, this study constructed a mediation effect model to explore the influencing mechanisms between health information literacy, vaccine hesitation, perceived vaccine information, and the intention to recommend COVID-19 vaccines. Table 1 shows the specific applications of the KAP theory and the proposed hypotheses.

### 2.2. Participants and Measures

This study combined existing established scales to design a questionnaire that included information on intention to recommend COVID-19 vaccination, health information literacy, vaccine hesitation, perceived vaccine information, and demographic characteristics. The questionnaire was validated by expert consultation and the opinions of the experts have passed the Delphi method test. Among them, the full score frequency *K_j_* of experts is above 0.8, which proves the validity and importance of the questionnaire [29]. A pilot study was conducted with 30 MBA students selected prior to the formal survey. Based on the pilot study, the descriptions of some of the questions were refined to ensure that the framework and information in the questionnaire were easy to understand and answer. Table 2 shows the measures of all variables. It took approximately 10–15 min to complete the questionnaire; thus, the length of the questionnaire was appropriate.

**Figure 1:**
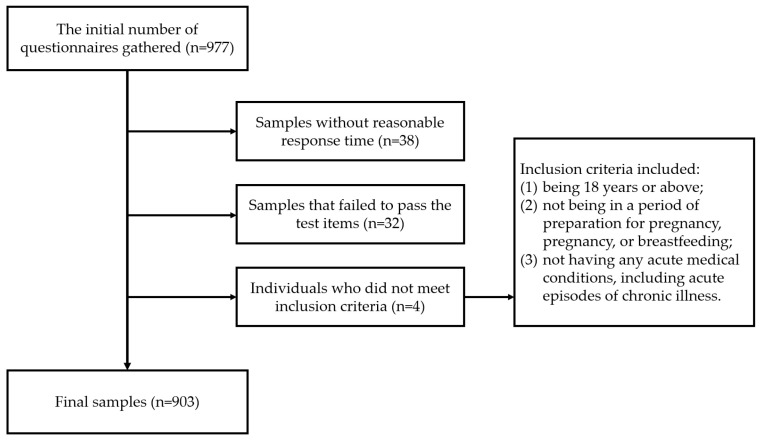
Procedures of sample selection.

A cross-sectional anonymous online survey of adults (aged > 18 years) was conducted in 28 provincial administrative regions of China in May 2022. The survey was carried out through a professional data collection platform (Credamo) in China. Figure 1 shows the procedures of sample selection. According to PASS16.0, a sample size of 903 achieved a minimum detectable odds ratio (OR) of 1.24 to 1.43 (power of 0.90 and alpha of 0.05, two-sided), assuming a frequency of intention to recommend the COVID-19 vaccination between 30% and 90%. Therefore, this study would have a good statistical power of at least 0.90 for associations with OR ≥ 1.24/1.43.

### 2.3. Data Analysis

This study used univariate logistic regression analysis to examine the crude association between background variables, influencing factors (i.e., health information literacy, safety hesitancy, effectiveness hesitancy, perceived vaccination information sufficiency, and perceived vaccination information usefulness), and two binary dependent variables (i.e., IRCVSG and IRCVNSG) and compared the differences between IRCVSG and IRCVNSG using the chi-square test. Subsequently, multivariate logistic regression analysis was used to adjust for background variables significantly associated with the dependent variable in the univariate analysis, and adjusted advantage ratios were calculated to assess the correlation between each influential factor and the two dependent variables. Finally, this study used path analysis with weighted least squares mean and variance adjusted estimates to test the hypothesized mediation model, adjusted for significant background variables. This study reported the direct and indirect effects of health information literacy on the dependent variable through safety hesitancy, vaccine efficacy hesitancy, perceived vaccination information sufficiency, and perceived vaccination information usefulness. The 95% bias-corrected confidence interval (CI) of indirect effects was estimated based on 2000 bootstrapped samples. The path’s standardized coefficients and statistical significance were displayed. The data of this study were analyzed using SPSS26.0 software. Two-sided *p* values below 0.05 were considered statistically significant.

## 3. Results

### 3.1. Demographics of Participants

A total of 903 participants were included for analysis (Table 3). Most of the participants were female (64.1%), were aged 18–40 (92%), received college or above education (95.9%), were married (69.1%), lived in eastern areas (70.3%), were frontline workers (46.8%), and had a monthly personal income above 5000 yuan (CNY) (75.2%).

### 3.2. Comparison of Intention to Recommend COVID-19 Vaccination for Different Groups

Overall, 840 (93.0%) of the participants reported an IRCVNS, while only 546 (60.5%) reported an IRCVSG (Table 3). The results of the chi-square test showed that there was significance in IRCVSG among different ages, marriage status, locations, occupations, and incomes. There was also significance in IRCVNSG among different ages and locations. The results of comparisons between IRCVSG and IRCVNSG showed that participants who were aged 18 to 40, were female, received above associate or bachelor education, lived in eastern areas, worked as management staff or on the frontline, and had a monthly personal income above 7000 yuan (CNY) had significantly different intentions between IRCVSG and IRCVNSG.

### 3.3. Distributions of the Independent Variables Associated with the IRCVSG/IRCVNSG

Table 4 shows the distributions of the factors associated with the IRCVSG and IRCVNSG, including health information literacy, safety hesitancy, effectiveness hesitancy, perceived vaccination information sufficiency, and perceived vaccination information usefulness. Using principal component analysis, the eight items of health information literacy were combined into health information literacy scores (Appendix A). The means and SDs of each item of health information literacy are also shown in Table 4. Among the participants, 19% (N = 171) had safety hesitancy for the COVID-19 vaccination, and 14% (N = 126) had effectiveness hesitancy for the COVID-19 vaccination. The mean levels of the perceived vaccination information sufficiency and perceived vaccination information usefulness were 3.98 (SD = 0.74) and 4.27 (SD = 0.77).

### 3.4. Crude Associations between the Background Variables and IRCVSG/IRCVNSG

Table 5 shows the results of the univariate logistic regression analysis. Important background factors for IRCVSG included age, marital status, location, occupation, and monthly personal income. People aged 31–40 (Orc = 1.572, 95% CI [1.186–2.084]), married (Orc = 2.342, 95% CI [1.756–3.125]) and higher-income groups have positive associations with IRCVSG, while people from the central region (Orc = 0.594, 95% CI [0.429–0.823]) and students (Orc = 0.395, 95% CI [0.272–0.574]) have negative associations with IRCVSG. The significant background factors for IRCVNSG included marriage status, occupation, and monthly personal income. Married (Orc = 2.158, 95% CI [1.288–3.615]) and high-income groups were positively associated with IRCVNSG, while students (Orc = 0.344, 95% CI [0.177–0.669]) were negatively associated with IRCVNSG.

### 3.5. Associations between the Independent Variables and IRCVSG/IRCVNSG

Table 6 shows the univariate logistic regression analysis results of independent variables for IRCVSG/IRCVNSG and the multivariate logistic regression results after adjusting for all other factors, including health information literacy, safety hesitancy, effectiveness hesitancy, perceived vaccination information sufficiency, and perceived vaccination information usefulness. Health information literacy was a significant factor of IRCVSG (ORa = 1.698, 95% CI [1.392–2.072]) and IRCVNSG (ORa = 2.365, 95% CI [0.22–0.59]). Safety hesitancy had significantly negative influence on IRCVSG (ORa = 0.468, 95% CI [0.331–0.663]) and IRCVNSG (ORa = 0.337,95% CI [0.196–0.577]), while effectiveness hesitancy had a significantly negative impact mainly on IRCVNSG (ORa = 0.218, 95% CI [0.125–0.380]). Perceived vaccination information sufficiency and perceived vaccination information usefulness were significantly positively associated with IRCVSG (ORa = 1.798, 95% CI [1.465–2.205]; ORa = 2.531, 95% CI [1.881–3.405]) and IRCVNSG (ORa = 1.683, 95% CI [1.388–2.039]; ORa = 3.791, 95% CI [2.733–5.269]).

### 3.6. Mediation Model of IRCVSG and IRCVNSG

**Figure 2:**
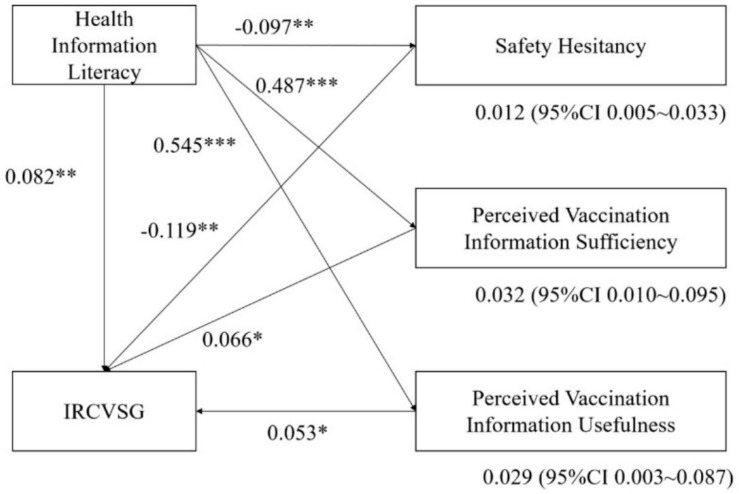
Proposed mediation model of the association between health information literacy and IRCVSG (N = 903). Note. CI, confidence interval; *** *p* < 0.001, ** *p* < 0.01, * *p* < 0.05.

The IRCVSG mediation model proposed in this study was shown in Figure 2. First, health information literacy had a significant positive direct effect on IRCVSG (standardized coefficient = 0.082, *p* < 0.01). Second, health information literacy had a significant positive effect on perceived vaccination information sufficiency (standardized coefficient = 0.487, *p* < 0.001) and perceived vaccination information usefulness (standardized coefficient = 0.545, *p* < 0.001), respectively, while perceived vaccination information sufficiency (standardized coefficient = 0.066, *p* < 0.05) and perceived vaccination information usefulness (standardized coefficient = 0.053, *p* < 0.05) had a significant positive effect on perceived vaccination information sufficiency (standardized coefficient = 0.066, *p* < 0.05) and perceived vaccination information usefulness (standardized coefficient = 0.053, *p* < 0.05) toward IRCVSG. Health information literacy had a significant positive indirect effect on IRCVSG through perceived vaccination information sufficiency (standardized coefficient = 0.032, 95% CI [0.010–0.095]) and perceived vaccination information usefulness (standardized coefficient = 0.029, 95% CI [0.003–0.087]). Third, health information literacy had a significant negative effect on safety hesitancy (standardized coefficient = −0.097, *p* < 0.001), respectively, while safety hesitancy (standardized coefficient = −0.119, *p* < 0.001) had a significant negative effect on IRCVSG. Health information literacy had a significant positive indirect effect on IRCVSG by weakening safety hesitancy (standardized coefficient = 0.012, 95% CI [0.005–0.033]). Figure 1 shows the standardized coefficients and statistical significance of the model pathways.

**Figure 3:**
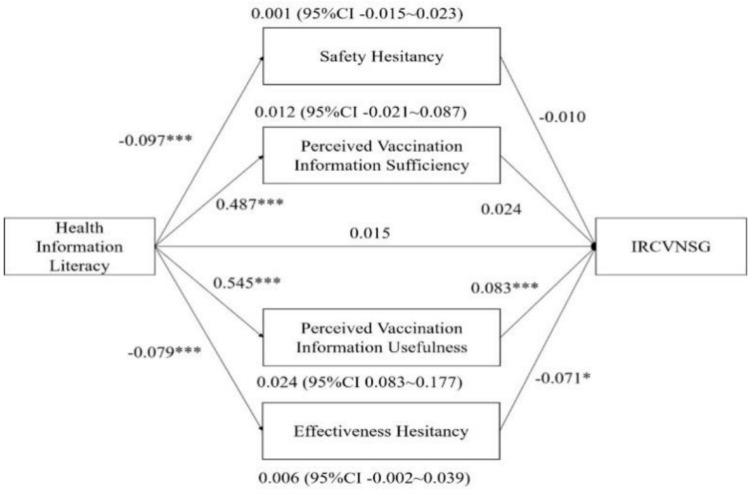
Proposed mediation model of the association between health information literacy and IRCVNSG (N = 903). Note. CI, confidence interval; *** *p* < 0.001, * *p* < 0.05.

In the mediated model of IRCVNSG, the indirect and direct effects of health information literacy differed slightly from the IRCVSG model (Figure 3). First, health information literacy had a significant positive effect on perceived vaccination information sufficiency (standardized coefficient = 0.487, *p* < 0.001) and perceived vaccination information usefulness (standardized coefficient = 0.545, *p* < 0.001), respectively. Perceived vaccination information usefulness (standardized coefficient = 0.083, *p* < 0.05) had a significant positive effect on IRCVNSG but perceived vaccination information usefulness (standardized coefficient = 0.053, *p* < 0.05) had an insignificant effect on IRCVNSG. Health information literacy had a significant positive indirect effect on IRCVSG through perceived vaccination information usefulness (standardized coefficient = 0.029, 95% CI [0.003–0.087]). Second, health information literacy had a significant negative effect on safety hesitancy (standardized coefficient = −0.097, *p* < 0.001) and effectiveness hesitancy (standardized coefficient = −0.079, *p* < 0.001), respectively. Effectiveness hesitancy (standardized coefficient = −0.071, *p* < 0.001) had a significant negative effect on IRCVNSG, while vaccine effectiveness hesitancy (standardized coefficient = −0.010, *p* > 0.05) had an insignificant effect on IRCVNSG. Health information literacy had a significant positive indirect effect on IRCVSG by enhancing perceived vaccination information usefulness and weakening effectiveness hesitancy (standardized coefficient = 0.012, 95% CI [0.005–0.033]). In addition, the direct effect of health information literacy on IRCVNSG was not significant (standardized coefficient = 0.015, *p* > 0.05). It suggested that health information literacy influences IRCVNSG exclusively through perceived vaccination information usefulness and effectiveness hesitation.

## 4. Discussion

Based on the KAP theory, this study investigated the relationship between health information literacy, vaccine hesitancy, perceived vaccination information, and intention to recommend COVID-19 vaccination to others in the Chinese population. The findings suggested that people with higher health information literacy, lower hesitancy in vaccine safety and effectiveness, and greater perceived vaccination information sufficiency and usefulness were more likely to recommend the COVID-19 vaccination to others. Additionally, this study further explored two major mechanisms by which health information literacy affected the intention to recommend vaccination.

Compared to existing studies, this study makes the following three significant contributions. First, this study is the first to differentiate the intention to recommend vaccines according to the specificity, which has implications for the development of targeted vaccine promotion policies and advocacy strategies for different groups. Second, this study reveals the effective mechanisms that affect the perceptions of vaccines, attitudes, and intention to recommend vaccines at the source, which adds a solid theoretical foundation to explain the generation of appropriate COVID-19 infection prevention behaviors. Finally, this study extends the knowledge dimension of the literacy dimension of the KAP model to health information, highlighting that the correct use of health information is more critical than the acquisition of knowledge in the context of the information epidemic. It extends the theoretical framework for analyzing the factors of intention to recommend vaccines [31,32] and provides new research insights to explain the emergence and changes in behavioral health decisions.

At the practical level, this study revealed that facing public health emergencies should not only focus on improving health knowledge but also direct focus to the cultivation of public health information literacy and the development of health information services. Previous research has shown that people with low levels of health information literacy are more susceptible to misinformation about COVID-19 vaccination and are unable to make good decisions [10,33]. Our findings build on this and demonstrate that health information literacy is fundamental to health behavior decisions. It complemented the KAP theory.

From a health communication and health behavior perspective, we need to eliminate misinformation in vaccination and outreach to help people reduce vaccine hesitancy and increase their perception of vaccination information, thereby increasing their intentions to recommend COVID-19 vaccination. This study found that safety hesitation and effectiveness hesitation are important factors affecting the intention to recommend vaccination, which is consistent with the results of previous studies [34].

Furthermore, many studies have confirmed a significant negative association between health information literacy and concerns about vaccine safety and effectiveness [35,36]. Previous studies on influenza and human papillomavirus vaccination have shown that frequency of exposure to vaccination information is positively associated with the presence of positive beliefs about vaccines [37,38]. The results of this study also supported this view. Our findings further supported the positive impact of health information literacy on access to health information [20]. Additionally, we found that improved perceived vaccination information sufficiency and perceived vaccination information usefulness increased intention to recommend the COVID-19 vaccination. This was similar to previous studies related to vaccination [39,40,41]. These findings revealed the need for governments to improve public perception of vaccination information in promotion. Experiments have shown that the intentions to recommend vaccines increase significantly after implementing interventions to improve perceived information usefulness, such as reading academic articles about vaccines, watching interviews with physicians, and reading institutional leaflets on virus prevention [42]. In addition, it is also very important to obtain specific information about antibodies after vaccination, such as different response data at 3 months and 6 months after vaccination [43], which can help the community build health information literacy. Therefore, government and medical institutions can build online platforms that provide health information and support services with the help of professional teams to ensure that the public has access to professional advice and the necessary information support on vaccines to enhance the intention to recommend COVID-19 vaccination.

An interesting finding of this study is that weakening hesitancy about the safety of vaccination can more effectively address vaccination dilemmas in specific groups in clinical practice. This has also been proved in the related research of low- and middle-income country [44]. The results showed that safety hesitation, perceived sufficiency of vaccination information, and perceived usefulness of vaccination information played a mediating role in the recommendation of COVID-19 vaccination to a specific group, while the impact of safety hesitation was not significant in the recommendation to a non-specific group. Previous studies have reported that parents are more concerned about the safety of vaccines than the effectiveness of vaccines when vaccinating their children [23]. This study further shows that the public needs to pay more attention to the safety of COVID-19 vaccination when recommending it to specific groups and make recommendations based on full understanding of relevant information. This means that proof of vaccine safety is essential to vaccinate specific groups against COVID-19. Future studies should further demonstrate that COVID-19 vaccination poses a low health risk to specific groups, and provide timely data on the safety, effectiveness, and public health impact of COVID-19 vaccination. The implications for workplace health and work sustainability are highlighted.

There are some limitations in this study. First, this study uses a cross-sectional study design, so the path model has limited ability to explain causality between variables. Future longitudinal study methods need to be designed to further verify causality. Second, participants may not be acting out their true intentions due to social expectations, leading to a bias in self-reported results. To ensure the confidentiality of information, the questionnaire of this study was answered anonymously by Chinese citizens on a network platform. Third, the universality of this study results needs to be further verified because this study was limited to the Chinese population, and factors such as cultural and religious beliefs in different countries may have an impact on the vaccine recommendations of the population.

## 5. Conclusions

In conclusion, from the perspective of health information literacy, this study investigated for the first time the influencing factors of Chinese adults’ intentions to recommend COVID-19 vaccines based on the KAP theory. The study found that health information literacy can reduce vaccine hesitation and enhance the perception of vaccine information, thereby increasing the intentions to recommend COVID-19 vaccines. In addition, when the recommended target group is a specific group, the public is relatively less willing to recommend COVID-19 vaccines and will pay more attention to vaccine safety. These findings remind us that in the context of the COVID-19 pandemic, improving people’s health information literacy is particularly important for vaccine promotion and herd immunity. Safety needs to be taken seriously when promoting vaccines to specific groups. In the future, scientific research institutions should develop safer and more effective COVID-19 vaccines for specific groups and conduct further research on the impact of group vaccine promotion and group immunity on health at the theoretical level.

## Figures and Tables

**Table 1:** Application of KAP theory for hypothesis development.

Dimension (Variables)	Application of KAP Theory	Hypotheses
**Knowledge Dimension** **(Health information literacy)**	In terms of the knowledge dimension, health information literacy helps people identify and judge the source and quality of information so that they can use it to make sound health decisions, including appropriate COVID-19 preventive behaviors [20,21,22,23].	**H1.** *Health information literacy has an impact on the intentions to recommend COVID-19 vaccination to specific groups*. **H2.** *Health information literacy influences the intentions to recommend COVID-19 vaccination to non-specific groups.*
**Attitude Dimension** **(Safety hesitancy and Effectiveness hesitancy)**	In the attitudinal dimension of the KAP theory, the effects of vaccine hesitation and vaccine information perception on vaccine recommendation intention are equally important [24,25] and play a mediating role between health information literacy and vaccine recommendation intention. Conversely, a lack of health information literacy, disrupted by misinformation on social media, may prevent people from forming correct knowledge about COVID-19 vaccines [21,22], thereby negatively impacting the intention to recommend COVID-19 vaccination.	**H3.** *Safety hesitation mediates the impact of health information literacy on the intentions to recommend COVID-19 vaccines to specific groups.* **H4.** *Safety hesitation mediates the effect of health information literacy on the intentions to recommend COVID-19 vaccines to non-specific groups.* **H5.** *Effectiveness hesitancy mediates the impact of health information literacy on the intentions to recommend COVID-19 vaccines to specific groups.* **H6.** *Effectiveness hesitation mediates the impact of health information literacy on the intentions to recommend COVID-19 vaccines to non-specific groups*.
**Attitude Dimension** **(Perceived vaccination information sufficiency and Perceived vaccination information usefulness)**	People expect effective and adequate perception of vaccination information to increase their trust in vaccines and engage in appropriate health behaviors [26,27]. People with high health information literacy tend to find more sufficient and useful information during information search [28].	**H7.** *Perceived adequacy of vaccination information mediates the impact of health information literacy on intentions to recommend COVID-19 to specific groups.* **H8.** *Perceived adequacy of vaccination information mediates the impact of health information literacy on intentions to recommend COVID-19 vaccines to non-specific groups.* **H9.** *Perceived usefulness of vaccination information mediates the impact of health information literacy on intentions to recommend COVID-19 vaccines to specific groups.* **H10.** *Perceived usefulness of vaccination information mediates the effect of health information literacy on the intentions to vaccinate non-specific groups for COVID-19.*

**Table 2:** Measures of variables.

Variables	Measures (Items)	Response Categories
Intention to recommend COVID-19 vaccination to specific groups	Would you recommend the COVID-19 vaccination to specific groups around you, for example, infants and children aged 6–23 months, pregnant women, older people over 60 years of age, people with chronic and immune-compromising diseases?	‘1 = definitely not’ to ‘5 = definitely yes’. Those who answered 4 or 5 were defined as having the intention.
Intention to recommend COVID-19 vaccination to non-specific groups	Besides the special groups mentioned above, would you recommend the COVID-19 vaccination to other people around you who meet the vaccination requirements?	‘1 = definitely not’ to ‘5 = definitely yes’. Those who answered 4 or 5 were defined as having the intention.
Health information literacy	Using scale developed by Norman and Skinner [30] with eight items: (1) I know how to find helpful health resources; (2) I know what health resources are available; (3) I know where to find helpful health resources; (4) I have the skills I need to evaluate health resources; (5) I can tell high-quality from low-quality health resources; (6) I know how to use health resources to answer health questions; (7) I know how to use health information to help myself and others; (8) I feel confident in using health information to make health decisions. The results of the reliability test showed that Cronbach’s α = 0.859, indicating that the reliability of this scale is satisfactory.	‘1 = strongly disagree’ to ‘5 = strongly agree’
Safety hesitancy	Would you be reluctant to recommend that others around you receive the COVID-19 vaccine because of concerns about the safety of the vaccine?	‘1 = yes’ and ‘0 = no’
Effectiveness hesitancy	Would you be reluctant to recommend that others around you receive the COVID-19 vaccine because of concerns about the effectiveness of the vaccine?	‘1 = yes’ and ‘0 = no’
Perceived vaccination information sufficiency	Do you think the information you have currently received about COVID-19 vaccination is sufficient for you to decide whether to recommend the COVID-19 vaccination?	‘1 = very insufficient’, ‘2 = slightly insufficient’, ‘3 = neutral’, ‘4 = slightly sufficient’, and ‘5 = very sufficient’
Perceived vaccination information usefulness	Do you think the information you currently received about COVID-19 vaccination is useful for you to decide whether to recommend the COVID-19 vaccination?	‘1 = very useless’, ‘2 = slightly useless’, ‘3 = neutral’, ‘4 = slightly useful’, and ‘5 = very useful’
Background information	Sociodemographic characteristics included gender, age (18–30; 31–40; 41–50; >50), level of education (senior high school or below; associate or bachelor; master or above), marriage status (married; single, divorced or widowed), occupation (frontline workers; management staff; self-employed; unemployed; students), area of residence (eastern; central; western), the average monthly personal income (<3000; 3000~4999; 5000~6999; 7000~9999; ≥10,000 ¥)	

**Table 3:** Demographic characteristics of the study population (N = 903).

	Overall N (%)	IRCVSG, N (%)		IRCVNSG, N (%)		Comparison between IRCVSG and IRCVNSG	
		Yes, N = 546 (60.5)	No, N = 357 (39.5)	Yes, N = 840 (93.0)	No, N = 63 (7.0)	$\chi^{2}$ (Overall = 23.367)	*p* Value (Overall < 0.001 ***)
Age		$\chi^{2}$ = 13.158, *p* = 0.004 **		$\chi^{2}$ = 2.260, *p* = 0.520			
18–30	438 (48.5)	244 (55.7)	194 (44.3)	402 (91.8)	36 (8.2)	6.105	0.013 *
31–40	393 (43.5)	261 (66.4)	132 (33.6)	370 (94.1)	23 (5.9)	21.857	<0.001 ***
41–50	45 (5.0)	29 (64.4)	16 (35.6)	42 (93.3)	3 (6.7)	0.007	0.934
>50	27 (3.0)	12 (44.4)	15 (55.6)	26 (96.3)	1 (3.7)	0.831	0.362
Sex		$\chi^{2}$ = 0.170, *p* = 0.680		$\chi^{2}$ = 0.426, *p* = 0.514			
Male	324 (35.9)	193 (59.6)	131 (40.4)	299 (92.3)	25 (7.7)	6.248	0.012 *
Female	579 (64.1)	353 (61.0)	226 (39.0)	541 (93.4)	38 (6.6)	17.522	<0.001 ***
Education		$\chi^{2}$ = 3.529, *p* = 0.171		$\chi^{2}$ = 0.223, *p* = 0.895			
Senior high school or below	37 (4.1)	19 (51.4)	18 (48.6)	35 (94.6)	2 (5.4)	2.003	0.157
Associate or bachelor	707 (78.3)	422 (59.7)	285 (40.3)	658 (93.1)	49 (6.9)	18.499	<0.001 ***
Master or above	159 (17.6)	105 (66.0)	54 (34.0)	147 (92.5)	12 (7.5)	9.746	0.002 **
Marriage status		$\chi^{2}$ = 34.194, *p* < 0.001 ***		$\chi^{2}$ = 8.870, *p* = 0.003 **			
Others (single, divorced or widowed)	279 (30.9)	129 (46.2)	150 (53.8)	249 (89.2)	30 (10.8)	5.179	0.023 *
Married	624 (69.1)	417 (66.8)	207 (33.2)	591 (94.7)	33 (5.3)	14.586	<0.001 ***
Location		$\chi^{2}$ = 11.046, *p* = 0.004 **		$\chi^{2}$ = 3.785, *p* = 0.151			
Eastern	635 (70.3)	406 (63.9)	229 (36.1)	584 (92.0)	51 (8.0)	18.726	<0.001 ***
Central	193 (21.4)	99 (51.3)	94 (48.6)	185 (95.9)	8 (4.1)	0.028	0.868
Western	75 (8.3)	41 (54.7)	34 (45.3)	71 (94.7)	4 (5.3)	1.870	0.171
Occupation		$\chi^{2}$ = 28.998, *p* < 0.001 ***		$\chi^{2}$ = 11.810, *p* = 0.019 *			
Frontline workers	423 (46.8)	285 (67.4)	138 (32.6)	404 (95.5)	19 (4.5)	5.780	0.016 *
Management staff	290 (32.1)	176 (60.7)	114 (39.3)	269 (92.8)	21 (7.2)	9.789	0.002 **
Self-employed	25 (2.8)	12 (48.0)	13 (52.0)	22 (88.0)	3 (12.0)	0.294	0.588
Unemployed	7 (0.8)	2 (28.6)	5 (71.4)	6 (85.7)	1 (14.3)	0.467	0.495
Student	158 (17.5)	71 (44.9)	87 (55.1)	139 (88.0)	19 (12.0)	3.026	0.082
Monthly personal income (¥)		$\chi^{2}$ = 28.349, *p* < 0.001 ***		$\chi^{2}$ = 9.632, *p* = 0.047 *			
<3000	139 (15.4)	61 (43.9)	78 (56.1)	121 (87.1)	18 (12.9)	2.178	0.140
3000~4999	85 (9.4)	43 (50.6)	42 (49.4)	81 (95.3)	4 (4.7)	0.001	0.981
5000~6999	115 (12.7)	70 (60.9)	45 (39.1)	108 (93.9)	7 (6.1)	1.015	0.314
7000~9999	213 (23.6)	133 (62.4)	80 (37.6)	202 (94.8)	11 (5.2)	6.117	0.013 *
≥10,000	351 (38.9)	239 (68.1)	112 (31.9)	328 (93.4)	23 (6.6)	16.063	<0.001 ***

Note. IRCVSG: the intention to recommend COVID-19 vaccination to specific groups; IRCVNSG: the intention to recommend COVID-19 vaccination to non-specific groups; *** *p* < 0.001, ** *p* < 0.01, * *p* < 0.05.

**Table 4:** Distributions of the independent variables associated with the IRCVSG/IRCVNSG (N = 903).

Variables	Mean	S.D.
1. Health information literacy (1 = totally disagree–5 = totally agree)	NA	NA
1.1 I know how to find helpful health resources.	4.16	0.66
1.2 I know what health resources are available.	4.19	0.67
1.3 I know where to find helpful health resources.	4.19	0.72
1.4 I have the skills I need to evaluate health resources.	3.91	0.86
1.5 I can tell high-quality from low-quality health resources.	3.98	0.76
1.6 I know how to use health resources to answer health questions.	4.13	0.73
1.7 I know how to use health information to help myself and others.	4.17	0.69
1.8 I feel confident in using health information to make health decisions.	4.08	0.79
2. Safety hesitancy (1 = yes, 0 = no)	0.19	0.39
3. Effectiveness hesitancy (1 = yes, 0 = no)	0.14	0.35
4. Perceived vaccination information sufficiency (1 = totally disagree–5 = totally agree)	3.98	0.74
5. Perceived vaccination information usefulness (1 = totally disagree–5 = totally agree)	4.27	0.77

Note. S.D., standard deviation; IRCVSG: the intention to recommend COVID-19 vaccination to specific groups; IRCVNSG: the intention to recommend COVID-19 vaccination to non-specific groups.

**Table 5:** Crude associations between the background variables and IRCVSG/IRCVNSG (N = 903).

	IRCVSG		IRCVNSG	
	ORc (95% CI) ^a^	*p* Value	ORc (95% CI) ^a^	*p* Value
*Age*				
18–30	(Reference group)			
31–40	1.572 (1.186–2.084)	0.002 **	1.441 (0.838–2.477)	0.187
41–50	1.441 (0.761–2.730)	0.262	1.254 (0.370–4.246)	0.716
>50	0.636 (0.291–1.391)	0.257	2.328(0.30717.661)	0.414
*Sex*				
Male	(Reference group)			
Female	1.060 (0.803–1.400)	0.680	1.190 (0.705–2.010)	0.515
*Education*				
Senior high school or below	(Reference group)			
Associate or bachelor	1.403 (0.724–2.719)	0.316	0.767 (0.179–3.285)	0.721
Master or above	1.842 (0.894–3.797)	0.098	0.700 (0.150–3.271)	0.650
*Marriage Status*				
Others (single, divorced or widowed)	(Reference group)			
Married	2.342 (1.756–3.125)	<0.001 ***	2.158 (1.288–3.615)	0.003 **
*Location*				
Eastern	(Reference group)			
Central	0.594 (0.429–0.823)	0.002 **	2.019 (0.941–4.333)	0.071
Western	0.680 (0.420–1.102)	0.118	1.550 (0.544–4.417)	0.412
*Occupation*				
Frontline workers	(Reference group)			
Management staff	0.748 (0.548–1.020)	0.067	0.602 (0.318–1.142)	0.120
Self-employed	0.447 (0.199–1.005)	0.051	0.345 (0.095–1.254)	0.106
Unemployed	0.194 (0.037–1.011)	0.052	0.282 (0.032–2.463)	0.252
Student	0.395 (0.272–0.574)	<0.001 ***	0.344 (0.177–0.669)	0.002 **
*Monthly personal income (¥)*				
<3000	(Reference group)			
3000~4999	1.309 (0.762–2.249)	0.329	3.012 (0.984–9.227)	0.053
5000~6999	1.989 (1.203–3.288)	0.007 **	2.295 (0.923–5.706)	0.074
7000~9999	2.126 (1.376–3.284)	<0.001 ***	2.732 (1.248–5.978)	0.012 *
≥10,000	2.729 (1.823–4.084)	<0.001 ***	2.121 (1.106–4.068)	0.024 *

Notes. CI, confidence interval; ^a^ ORc: crude odds ratio obtained by using univariate logistic regression on the binary intention to recommend COVID-19 vaccinations to specific/non-specific groups was performed for each independent variable; IRCVSG: the intention to recommend COVID-19 vaccination to specific groups; IRCVNSG: the intention to recommend COVID-19 vaccination to non-specific groups; *** *p* < 0.001, ** *p* < 0.01, * *p* < 0.05.

**Table 6:** Adjusted associations between the influencing factors and the IRCVSG/IRCVNSG (N = 903).

Variables	IRCVSG, ORc (95% CI) ^a^	IRCVNSG, ORc (95% CI) ^a^	IRCVSG, ORa (95% CI) ^b^	IRCVNSG, ORa (95% CI) ^b^
Health Information Literacy	1.925 (1.592–2.328) ***	2.546 (1.907–3.398) ***	1.698 (1.392–2.072) ***	2.365 (1.743–3.207) ***
Safety Hesitation	0.434 (0.310–0.606) ***	0.307 (0.180–0.522) ***	0.468 (0.331–0.663) ***	0.337 (0.196–0.577) ***
Effectiveness Hesitation	0.743 (0.510–1.081)	0.238 (0.108–0.523) ***	0.754 (0.509–1.117)	0.218 (0.125–0.380) ***
Perceived Vaccination Information Sufficiency	1.928 (1.583–2.347) ***	2.694 (2.024–3.585)***	1.798 (1.465–2.205)***	2.531 (1.881–3.405) ***
Perceived Vaccination Information Usefulness	1.815 (1.510–2.182) ***	3.945 (2.863–5.436) ***	1.683 (1.388–2.039) ***	3.791 (2.733–5.269) ***

Note: CI, confidence interval; ^a^ ORc: crude odds ratio obtained by using univariate logistic regression on the binary intention to recommend COVID-19 vaccinations to specific/non-specific groups was performed for each independent variable; ^b^ ORa: adjusted odds ratio obtained by using multivariable logistic regression on the binary intention to recommend COVID-19 vaccinations to specific/non-specific groups was performed for each independent variable, with age, sex, education, marriage status, location, occupation and monthly personal income; IRCVSG: the intention to recommend COVID-19 vaccination to specific groups; IRCVNSG: the intention to recommend COVID-19 vaccination to non-specific groups; *** *p* < 0.001.

## Data Availability

The datasets generated during the current study are available from the corresponding author on reasonable request.

## Appendix A

**Table A1:** KMO and Bartlett’s Test.

**KMO**		0.895
**Bartlett’s test of sphericity**	Approx. chi-square	2537.938
	df	28
	*p* value	<0.001

**Table A2:** Total variance explained.

Factor	Eigen			Principle Component Extraction		
	Eigenvalue	% of Variance	Cumulative % of Variance	Eigenvalue	% of Variance	Cumulative % of Variance
**1**	4.040	50.501	50.501	4.040	50.501	50.501
**2**	0.836	10.445	60.946	-	-	-
**3**	0.693	8.663	69.609	-	-	-
**4**	0.625	7.809	77.418	-	-	-
**5**	0.526	6.571	83.989	-	-	-
**6**	0.470	5.876	89.866	-	-	-
**7**	0.414	5.174	95.040	-	-	-
**8**	0.397	4.960	100.000	-	-	-

**Table A3:** Component score coefficient matrix.

Name	Component
	Component 1
**HL1**	0.168
**HL2**	0.174
**HL3**	0.163
**HL4**	0.178
**HL5**	0.171
**HL6**	0.183
**HL7**	0.172
**HL8**	0.194

**Health information literacy scores** = 0.168 × HL1 + 0.174 × HL2 + 0.163 × HL3 + 0.178 × HL4 + 0.171 × HL5 + 0.183 × HL6 + 0.172 × HL7 + 0.194 × HL8.
